# Burden of in-hospital care in oesophageal cancer: national population-based study

**DOI:** 10.1093/bjsopen/zrab037

**Published:** 2021-05-07

**Authors:** G Linder, F Klevebro, D Edholm, J Johansson, M Lindblad, J Hedberg

**Affiliations:** Department of Surgical Sciences, Uppsala University, Uppsala, Sweden; Department of Clinical Science, Intervention and Technology, Centre for Upper Gastrointestinal Cancer, Karolinska University Hospital, Karolinska Institutet, Stockholm, Sweden; Department of Surgery, Biomedical and Clinical Sciences, Linköping University, Linköping, Sweden; Department of Surgery, Lund University, Lund, Sweden; Department of Clinical Science, Intervention and Technology, Centre for Upper Gastrointestinal Cancer, Karolinska University Hospital, Karolinska Institutet, Stockholm, Sweden; Department of Surgical Sciences, Uppsala University, Uppsala, Sweden

## Abstract

**Background:**

Oesophageal cancer management requires extensive in-hospital care. This cohort study aimed to quantify in-hospital care for patients with oesophageal cancer in relation to intended treatment, and to analyse factors associated with risk of spending a large proportion of survival time in hospital.

**Methods:**

All patients with oesophageal cancer in three nationwide registers over a 10-year period were included. In-hospital care during the first year after diagnosis was evaluated, and the proportion of survival time spent in hospital, stratified by intended treatment (curative, palliative or best supportive care), was calculated. Associations between relevant factors and a greater proportion of survival time in hospital were analysed by multivariable logistic regression.

**Results:**

In-hospital care was provided for a median of 39, 26, and 15 days in the first year after diagnosis of oesophageal cancer in curative, palliative, and best supportive care groups respectively. Patients receiving curatively intended treatment spent a median of 12 per cent of their survival time in hospital during the first year after diagnosis, whereas those receiving palliative or best supportive care spent 19 and 23 per cent respectively. Factors associated with more in-hospital care included older age, female sex, being unmarried, and chronic obstructive pulmonary disease.

**Conclusion:**

The burden of in-hospital care during the first year after diagnosis of oesophageal cancer was substantial. Important clinical and socioeconomic factors were identified that predisposed to a greater proportion of survival time spent in hospital.

## Teaser

This study investigated in-hospital care during the first year after diagnosis of oesophageal cancer. The results provide a sobering picture of how large a proportion of remaining days alive are spent in hospital for subgroups of these patients. Factors associated with risk of spending a larger proportion of remaining life in hospital are also described.

## Introduction

Cancers affecting the oesophagus and gastro-oesophageal junction carry high mortality rates, causing up to 1 100 000 deaths per year globally^1–3^. Although the highest incidence is found in Asia, there has been a marked increase in incidence of adenocarcinoma in the West in recent decades. Advances in surgical techniques, perioperative care, and oncological adjuncts have increased the chance of curing localized disease^4^^,^^5^. In Sweden, healthcare is funded publicly and organized in 21 separate counties, with independent healthcare systems that refer patients to some six tertiary centres for management of oesophageal cancer. Nationwide consensus guidelines govern diagnostics, treatment, and follow-up. In 2018, more than 90 per cent of patients with oesophageal cancer were discussed in a multidisciplinary team conference. As in many European countries, curative treatment is predominantly multimodal^4^^,^^5^ and can be offered to around one-third of patients, whereas the greater proportion, diagnosed at an advanced stage, can only be offered palliation or best supportive care.

All patients diagnosed with cancer of the oesophagus or gastro-oesophageal junction require healthcare, including varying amounts of in-hospital care. For some, a large proportion of the remaining life after diagnosis is spent in hospital. The burden of in-hospital care can vary widely, mainly depending on the treatment offered. Information on this burden is scarce, although the number of hospital admissions for patients with gastric cancer in a Western setting has been described recently^6^. Nationwide data from the Swedish National Register for Oesophageal and Gastric Cancer (NREV) reported an overall 5-year survival rate of just 15 per cent for patients diagnosed with oesophageal cancer, emphasizing the importance of maintaining health-related quality of life, rather than offering futile interventions^7^.

A unique personal identification number, given to each citizen at birth in Sweden, makes it possible to link data from several registers. One such well validated register, the NREV, contains data on diagnosis and care, including information on intended treatment (curative, palliative, best supportive care), for all patients with these diagnoses since 2006. The National Patient Register (NPR) covers all diagnosis codes as well as dates of admission and discharge for inpatient care from 1987, and since 2001 has also covered outpatient clinic visits from public, as well as private, caregivers^8^. The Swedish Prescribed Drugs Register contains data on prescribed and patient-collected drugs since 2005, providing updated data on treatment patterns and co-morbidities.

The aim of the present study was to describe the total burden of in-hospital care for patients diagnosed with oesophageal cancer (including cancers of the oesophagogastric junction) over a 10-year interval, and to identify factors influencing the proportion of survival time spent in hospital during the first year after diagnosis.

## Methods

This study was approved by the regional ethical review board in Stockholm (2013/596–31/3 and 2016/1486–32). It included all patients with oesophageal cancer diagnosed in Sweden between 1 January 2006 and 31 December 2015. The primary selection base was the NREV^9^, including both adenocarcinoma and squamous cell carcinoma. Subsequently, patients not reported to NREV were added by searching the NPR for the diagnosis oesophageal cancer (ICD10 C15.0–15.9), including Siewert I and II tumours (ICD10 C16.0A, 16.0 B, C16.0X). Only patients registered in the Swedish Cancer Register were included. Individual-patient data were cross-linked with the Swedish Prescribed Drug Register^10^^,^^11^, Cause of Death Register^12^, Swedish Cancer Register^13^, and the NPR; additional demographic data as well as marital status and educational data were obtained from the Longitudinal Integrated Database for Health Insurance and Labour Market Studies from the Swedish National Board of Health and Welfare. Intended treatment (curative, palliative or best supportive care) was determined, for the vast majority of patients, by the multidisciplinary team after staging investigations or by the treating physician, and reported to the NREV as a mandatory variable. All the above registries are well described, researched, and validated^8–14^.

### Baseline clinical data

Data on sex, age at time of diagnosis, tumour location, and intended treatment were retrieved from the NREV. Information on co-morbidities at the time of diagnosis was compiled from the NPR and the Prescribed Drug Register, where diagnosis codes (according to ICD-10) were complemented by medication codes (according to the Anatomical Therapeutic Chemical (ATC) classification system). Patients with diabetes were defined as those taking non-insulin diabetic drugs (ATC A10B) and/or prescribed insulin treatment (ATC A10A). Chronic obstructive pulmonary disease (COPD) was defined by a diagnosis of COPD (ICD10 J44) or prescriptions for medications related to obstructive pulmonary disease (ATC R03A, R03B, R03C). Heart disease was defined by the patient having a diagnosis of heart failure (ICD10 I50) or coronary disease (ICD10 I21). Peripheral vascular disease was identified by diagnosis (ICD10 I73.9), or by drugs for vascular disease (ATC B01AC06) or hypertension (ATC C03, C07, C08, C09).

### Outcome

All episodes of in-hospital care in Sweden, regardless of hospital setting, during the first year after the diagnosis (day of diagnostic biopsy) were extracted and combined, giving a total number of days in hospital. The data extracted on in-hospital care included all inpatient care in all hospital settings, but excluded community-based home care. The proportion of time alive spent in hospital during the first year was calculated by dividing the number of days spent in hospital by number of days alive during the first year after the date of diagnosis (maximum value 365 days).

### Statistical analysis

Median overall survival was determined according to intended treatment. Median and total number of days of in-hospital care and annual amount per 100 000 inhabitants were calculated. The proportion of days alive spent in a hospital setting during the first year after diagnosis was calculated as a median value and visualized in violin plots to display the full distribution of proportions (kernel density) stratified by treatment intent. A directed acyclic graph model^15^ was used to identify possible factors governing outcome as well as confounders. Possible associations between identified factors and spending a relatively greater proportion (above median) of survival time in hospital during the first year after diagnosis was analysed by multivariable logistic regression, with results presented as adjusted odds ratios and 95 per cent confidence intervals. The multivariable model included age, sex, intended treatment (curative, palliative, best supportive care), year of diagnosis, clinical stage, education level, marital status, and co-morbidities (diabetes, COPD, heart disease, peripheral vascular disease).

## Results

A total of 6400 patients were identified. Patient characteristics, tumour location, clinical stage, sociodemographic details, co-morbidities, and intended treatment are outlined in *Table 1*.

**Table 1:** Characteristics of 6400 patients diagnosed with oesophageal or gastro-oesophageal junctional cancer between 2006 and 2015

	**No. of patients** **(*n* = 6400)**
**Age (years)**	
<60	1048 (16.4)
60–70	2006 (31.3)
70–80	1902 (29.7)
>80	1444 (22.6)
**Sex ratio (F : M)**	1671 : 4729
**Year of diagnosis**	
2006–2009	2444 (38.2)
2010–2012	1848 (28.9)
2013–2016	2108 (32.9)
**Clinical stage** ^†^	
I	204 (3.2)
II	757 (11.8)
III	1457 (22.8)
IVa	410 (6.4)
IVb	1814 (28.3)
Missing	1758 (27.5)
**Marital status**	
Not married	3155 (49.3)
Married	3245 (50.7)
**Education level (years)**	
<10	2324 (36.3)
10–12	2342 (36.6)
>12	1734 (27.1)
**Co-morbidities**	
Diabetes	1056 (16.5)
COPD	1076 (16.8)
Cardiac disease	908 (14.2)
Peripheral vascular disease	3910 (61.1)
**Intended treatment**	
Curative treatment	2286 (35.7)
Palliative treatment	2651 (41.4)
Best supportive care	763 (11.9)
Undefined	700 (10.9)

Values in parentheses are percentages. ICD-10 codes forgastro-oesophageal junctional cancer are C16.0A, C16.0B, and C16.0X. COPD, chronic obstructive pulmonary disease. †TNM classification, eighth edition.

**Fig. 1:**
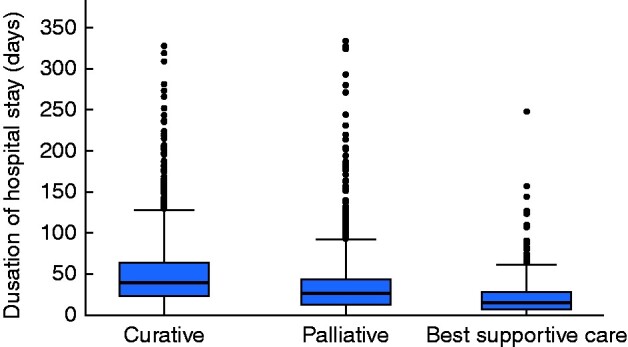
Box plots illustrating time spent in hospital care during the first year after diagnosis according to intended treatment for patients with oesophageal or gastro-oesophageal junctional cancer Median value (bold line), i.q.r. (box), and range (error bars) excluding outliers (circles) are shown.

Median overall survival was 23.9, 5.5, and 2.6 months in the curative, palliative, and best supportive care groups respectively. In-hospital care was provided for a median of 39, 26, and 15 days in these three groups for the first year after diagnosis (*Fig. 1*). This resulted in a total of 220 099 days of in-hospital care during the patient’s first year after diagnosis for the 10-year study, 115 657 (52.5 per cent) of which occurred in the group treated with curative intent. This total corresponded to 220 in-hospital days per 100 000 inhabitants per year, based on the Swedish population of 10 million inhabitants.

**Fig. 2:**
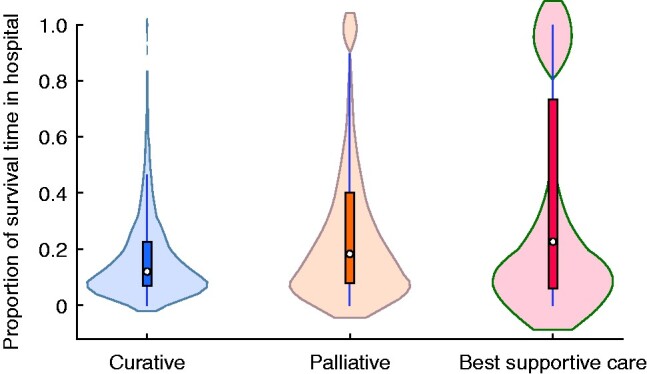
Violin plots illustrating the proportion of survival time spent in hospital in the first year after diagnosis according to intended treatment for oesophageal or gastro-oesophageal junctional cancer Median value (circle), i.q.r. (box), and lower to upper adjacent value (vertical line) are shown. The shaded area represents kernel density.

The median proportion of time alive spent in hospital during the first year after diagnosis was 12, 19, and 23 per cent in the curative, palliative, and best supportive care groups respectively (*Fig. 2*).

**Fig. 3:**
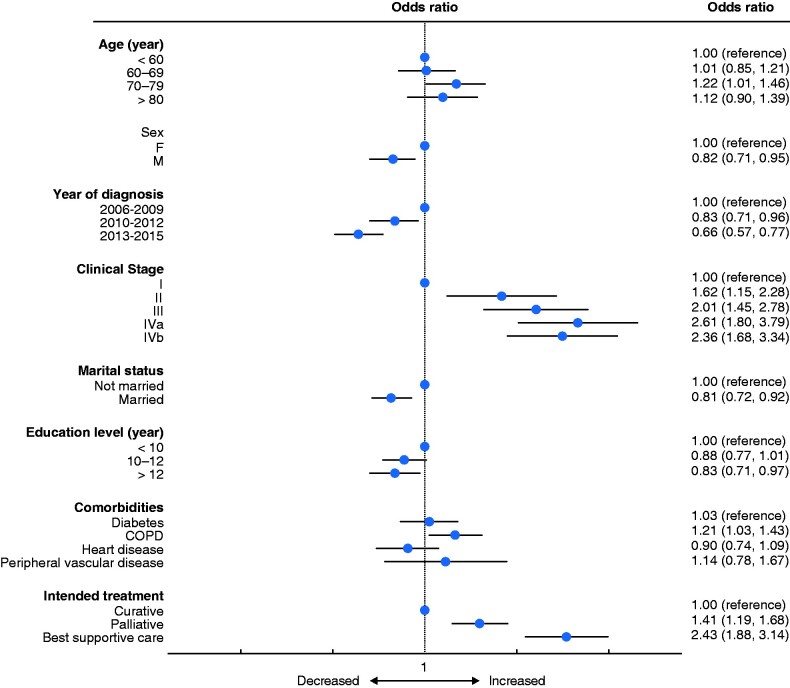
Results of multivariable logistic regression analysis for the outcome being above the median duration of in-hospital care during the first year after diagnosis of oesophageal or gastro-oesophageal junctional cancer. Odds ratios are shown with 95 per cent confidence intervals. COPD, chronic obstructive pulmonary disease.

In the multivariable logistic regression model, adjusting for co-variables previously identified by directed acyclic graph analysis as possible confounders, age above 70 years (*P* = 0.035), female sex (*P* = 0.006), more advanced tumour stage (*P* < 0.001), COPD (*P* = 0.024), and non-curative treatment intent (*P* < 0.001) all predisposed to a greater proportion of the patient’s remaining time alive being spent in hospital in the first year after diagnosis. High education level (*P* = 0.019), being married (*P* = 0.001), and a later calendar year of diagnosis (2010–2012 versus 2006–2009, *P* = 0.014 and 2013–2016 versus 2006–2009, *P* < 0.001) were all associated with spending a smaller proportion of remaining time alive in hospital (*Fig. 3*).

## Discussion

It has been estimated that oesophageal cancer accounts for 9.78 million disability-adjusted life-years (DALYs), with an age-standardized rate of 120 DALYs per 100 000 population, globally each year^1^. In Sweden, the age-standardized rate is lower (51 DALYs per 100 000 population)^2^. Estimates of age-standardized DALYs may be useful in appreciating disease burden to facilitate international comparisons, but provide little to support health economists on a national level. The estimated total annual burden of in-hospital care of 220 days per 100 000 population for patients with oesophageal cancer in the present study provides a better estimate, and can be used to study future impacts of public health interventions, such as smoking cessation and antiobesity-related campaigns. By comparison, the initial hospital admission rate for patients with pancreatic cancer in the USA was 50 per 100 000 person-years, with a mean duration of stay of 9.1 days in 2010^16^. This represents an annual in-hospital care burden of 455 days per 100 000 population, for initial admission only. For patients with oesophageal cancer, Sarvepalli and colleagues^17^ reported the annual hospitalization rate in 2010 to be [12(0.8) (mean(s.d.))] per 100 000 population with an average stay of [7.3(0.3) (mean(s.d.))] days. This equates to roughly 96 days of in-hospital care per 100 000 population for oesophageal cancer. A plausible reason for the higher burden of in-hospital care in the present study is that it includes all care in a hospital setting, not limited to surgical care, but also includes in-hospital care provided under any specialty.

Treatment protocols designed to achieve cure of oesophageal cancer are often multimodal, combining surgery with neoadjuvant chemotherapy or chemoradiotherapy. The resource-intense nature of this treatment explains why, in the present study, the greatest number of days in hospital occurred in the group undergoing potentially curative treatment (39 days). Patients receiving palliative or best supportive care had a shorter period of in-hospital care, but the proportion of remaining life spent in hospital was much greater. Although this accounted for only 12 per cent of the time in the first year after diagnosis in the curatively treated group, it amounted to 19 per cent of days alive for patients treated palliatively and 23 per cent for those receiving best supportive care. Some 139 (18.2 per cent) of the 763 patients receiving best supportive care remained in a hospital setting from the date of diagnosis until date of death. Although some of such patients need care that is available only in hospital, there is evidence that the majority want to be at home with family for end-of-life care^18^, and this can often be delivered successfully by community-based palliative care teams^19^. A systematic review^20^ provided strong evidence that home palliation or end-of-life care increased the chance of dying at home and reduced symptom burden, in particular for patients with cancer, without influencing caregiver grief. A better understanding of issues that contribute to quality of life among patients receiving palliative therapies or best supportive care might help to minimize the proportion of remaining life spent in hospital.

Although frailty in the elderly excludes some patients from anything other than supportive care, increasing age and advanced clinical disease stage had a clear association with a greater need for in-hospital care, as did being diagnosed during the early years of the study. The latter might reflect a general trend towards reducing duration of hospital stay as a result of multidisciplinary care and centralization of oesophageal cancer treatment to high-volume centres, along with more advanced home care, in the later years of the study.

Men had a reduced risk of spending a greater proportion time in hospital. Marital status, coping with the diagnosis, dealing with pain, and a reduced willingness among men to seek healthcare might all play a part^21^. Similar findings have been reported in colorectal cancer, where a recent Scandinavian study^22^ noted that married patients spent 30 days less in hospital, and used less home- and community-based care in an end-of-life setting. A study from 2019 found that married patients with advanced cancer were less likely to have potentially avoidable readmissions^23^. These findings highlight that spouses of married patients provide informal healthcare to a degree that can reduce the need for hospital admission.

Both curative treatment and high education level were associated with a decreased risk of spending a greater proportion of the first year after diagnosis in hospital. Patients with a higher education level are known to be more likely to receive curative treatment, and this level of education is associated with improved survival after curative treatment for oesophageal cancer^24^. As patients in the curative treatment group spent the lowest proportion of the first year in hospital (12 per cent), a co-variation between education level and curative treatment corroborates these previous findings.

Although pre-existing co-morbidities are known to influence the frequency and severity of postoperative complications among patients with oesophagogastric cancers^25^^,^^26^, only COPD was clearly associated with an increased risk of in-hospital care. That other investigated co-morbidities were not associated with risk of in-hospital care could be due to the aggressive nature of oesophageal cancer, as the majority of healthcare consumption after diagnosis relates to the management of symptoms of malignancy.

The register-based and retrospective design of the study has limitations. Some patients never actually received the intended curative treatment they were registered as having, and instead received only palliative or best supportive care. They were, however, handled on an intention-to-treat basis in this study. The registers also lacked potentially important information on the severity of co-morbidities and completeness of smoking status. Completeness of the variable clinical stage was low, owing to inability to assign an accurate clinical stage, according to the eighth edition of the TNM classification, to patients classified with any of Tx, Nx or Mx. Patients in the curative group undergoing neoadjuvant treatment appeared to have received more in-hospital care, not as a result of risk factors but rather an accumulation of in-hospital care owing to aggressive treatment of relatively fit patients. This may have clouded some associations by diluting the key findings of the multivariable analysis. Conversely, strengths of the study include its nationwide coverage and prospective collection of data to the NREV register, reducing the risk of selection bias. The Swedish population-based Cause of Death Register also ensured complete follow-up regarding survival.

## References

[1] GBD 2017 Oesophageal Cancer Collaborators. The global, regional, and national burden of oesophageal cancer and its attributable risk factors in 195 countries and territories, 1990–2017: a systematic analysis for the Global Burden of Disease Study 2017. Lancet Gastroenterol Hepatol 2020;5:582–597 32246941 10.1016/S2468-1253(20)30007-8 PMC7232026

[2] GBD 2017 Stomach Cancer Collaborators. The global, regional, and national burden of stomach cancer in 195 countries, 1990–2017: a systematic analysis for the Global Burden of Disease Study 2017. Lancet Gastroenterol Hepatol 2020;5:42–54 31648970 10.1016/S2468-1253(19)30328-0 PMC7033564

[3] Torre LA, Bray F, Siegel RL, Ferlay J, Lortet-Tieulent J, Jemal A. Global cancer statistics, 2012. CA Cancer J Clin 2015;65:87–108 25651787 10.3322/caac.21262

[4] Shapiro J, van Lanschot JJB, Hulshof M, van Hagen P, van Berge Henegouwen MI, Wijnhoven BPL et al; CROSS study group. Neoadjuvant chemoradiotherapy plus surgery *versus* surgery alone for oesophageal or junctional cancer (CROSS): long-term results of a randomised controlled trial. Lancet Oncol 2015;16:1090–1098 26254683 10.1016/S1470-2045(15)00040-6

[5] Al-Batran SE, Homann N, Pauligk C, Goetze TO, Meiler J, Kasper S et al Perioperative chemotherapy with fluorouracil plus leucovorin, oxaliplatin, and docetaxel *versus* fluorouracil or capecitabine plus cisplatin and epirubicin for locally advanced, resectable gastric or gastro-oesophageal junction adenocarcinoma (FLOT4): a randomised, phase 2/3 trial. Lancet 2019;393:1948–1957 30982686 10.1016/S0140-6736(18)32557-1

[6] Solanki S, Chakinala RC, Haq KF, Khan MA, Kifayat A, Linder K et al Inpatient burden of gastric cancer in the United States. Ann Transl Med 2019;7:772–772 32042788 10.21037/atm.2019.11.54 PMC6990011

[7] Merchant SJ, Brogly SB, Goldie C, Booth CM, Nanji S, Patel SV et al Palliative care is associated with reduced aggressive end-of-life care in patients with gastrointestinal cancer. Ann Surg Oncol 2018;25:1478–1487 29569126 10.1245/s10434-018-6430-9

[8] Ludvigsson JF, Andersson E, Ekbom A, Feychting M, Kim JL, Reuterwall C et al External review and validation of the Swedish national inpatient register. BMC Public Health 2011;11:450 21658213 10.1186/1471-2458-11-450 PMC3142234

[9] Linder G, Lindblad M, Djerf P, Elbe P, Johansson J, Lundell L et al Validation of data quality in the Swedish National Register for Oesophageal and Gastric Cancer. Br J Surg 2016;103:1326–1335. 27467590 10.1002/bjs.10234

[10] Wallerstedt SM, Wettermark B, Hoffmann M. The first decade with the Swedish Prescribed Drug Register—a systematic review of the output in the scientific literature. Basic Clin Pharmacol Toxicol 2016;119:464–469 27112967 10.1111/bcpt.12613

[11] Wettermark B, Hammar N, Fored CM, Leimanis A, Otterblad Olausson P, Bergman U et al The new Swedish Prescribed Drug Register—opportunities for pharmacoepidemiological research and experience from the first six months. Pharmacoepidemiol Drug Saf 2007;16:726–735 16897791 10.1002/pds.1294

[12] Brooke HL, Talback M, Hornblad J, Johansson LA, Ludvigsson JF, Druid H et al The Swedish cause of death register. Eur J Epidemiol 2017;32:765–773 28983736 10.1007/s10654-017-0316-1 PMC5662659

[13] Barlow L, Westergren K, Holmberg L, Talback M. The completeness of the Swedish Cancer Register: a sample survey for year 1998. Acta Oncol 2009;48:27–33 18767000 10.1080/02841860802247664

[14] Johansson LA, Westerling R. Comparing Swedish hospital discharge records with death certificates: implications for mortality statistics. Int J Epidemiol 2000;29:495–502 10869322

[15] VanderWeele TJ, Hernan MA, Robins JM. Causal directed acyclic graphs and the direction of unmeasured confounding bias. Epidemiology 2008;19:720–728 18633331 10.1097/EDE.0b013e3181810e29 PMC4242711

[16] Wang Y, Schrag D, Brooks GA, Dominici F. National trends in pancreatic cancer outcomes and pattern of care among Medicare beneficiaries, 2000 through 2010. Cancer 2014;120:1050–1058 24382787 10.1002/cncr.28537 PMC4019988

[17] Sarvepalli S, Garg SK, Sarvepalli SS, Parikh MP, Wadhwa V, Jang S et al Inpatient burden of esophageal cancer and analysis of factors affecting in-hospital mortality and length of stay. Dis Esophagus 2018;31: 1–7. 10.1093/dote/doy022 PMC7055505 29617798

[18] Gomes B, Calanzani N, Gysels M, Hall S, Higginson IJ. Heterogeneity and changes in preferences for dying at home: a systematic review. BMC Palliat Care 2013;12:7 23414145 10.1186/1472-684X-12-7 PMC3623898

[19] Fringer A, Stängle S, Bischofberger I, Büche D, Praxmarer R, Ch Ott S et al Experiences of relatives with outpatient palliative care: a cross-sectional study. Int J Palliat Nurs 2020;26:230–237 32584687 10.12968/ijpn.2020.26.5.230

[20] Gomes B, Calanzani N, Curiale V, McCrone P, Higginson IJ. Effectiveness and cost-effectiveness of home palliative care services for adults with advanced illness and their caregivers. Cochrane Database Syst Rev 2013;(6)CD007760 10.1002/14651858.CD007760.pub2 PMC4473359 23744578

[21] Mogil JS, Bailey AL. Sex and gender differences in pain and analgesia. Prog Brain Res 2010;186:141–157 21094890 10.1016/B978-0-444-53630-3.00009-9

[22] Bjørnelv GMW, Edwin B, Fretland ÅA, Deb P, Aas E. Till death do us part: the effect of marital status on health care utilization and costs at end-of-life. A register study on all colorectal cancer decedents in Norway between 2009 and 2013. BMC Health Serv Res 2020;20:115 32054492 10.1186/s12913-019-4794-6 PMC7020544

[23] Johnson PC, Xiao Y, Wong RL, D'Arpino S, Moran SMC, Lage DE et al Potentially avoidable hospital readmissions in patients with advanced cancer. J Oncol Pract 2019;15:e420–e427 30946642 10.1200/JOP.18.00595 PMC7846058

[24] Linder G, Sandin F, Johansson J, Lindblad M, Lundell L, Hedberg J. Patient education-level affects treatment allocation and prognosis in esophageal- and gastroesophageal junctional cancer in Sweden. Cancer Epidemiol 2018;52:91–98 29278841 10.1016/j.canep.2017.12.008

[25] Klevebro F, Elliott JA, Slaman A, Vermeulen BD, Kamiya S, Rosman C, Gisbertz SS et al Cardiorespiratory comorbidity and postoperative complications following esophagectomy: a European Multicenter Cohort Study. Ann Surg Oncol 2019;26:2864–2873 31183640 10.1245/s10434-019-07478-6 PMC6682565

[26] Coimbra FJF, de Jesus VHF, Franco CP, Calsavara VF, Ribeiro HSC, Diniz AL et al Predicting overall and major postoperative morbidity in gastric cancer patients. J Surg Oncol 2019;120:1371–1378 31696512 10.1002/jso.25743

